# Assessing Differential Particle Deformability under Microfluidic Flow Conditions

**DOI:** 10.1021/acsbiomaterials.3c00120

**Published:** 2023-05-17

**Authors:** Marco E. Miali, Wei Chien, Thomas Lee Moore, Alessia Felici, Anna Lisa Palange, Michele Oneto, Dmitry Fedosov, Paolo Decuzzi

**Affiliations:** † Laboratory of Nanotechnology for Precision Medicine, Fondazione Istituto Italiano di Tecnologia, Via Morego 30, 16163 Genoa, Italy; ‡ Institute of Biological Information Processing, Forschungszentrum Jülich GmbH, Wilhelm-Johnen-Straße, 52428 Jülich, Germany

**Keywords:** microfluidics, KOH wet etching, modeling, image processing, particle dynamics

## Abstract

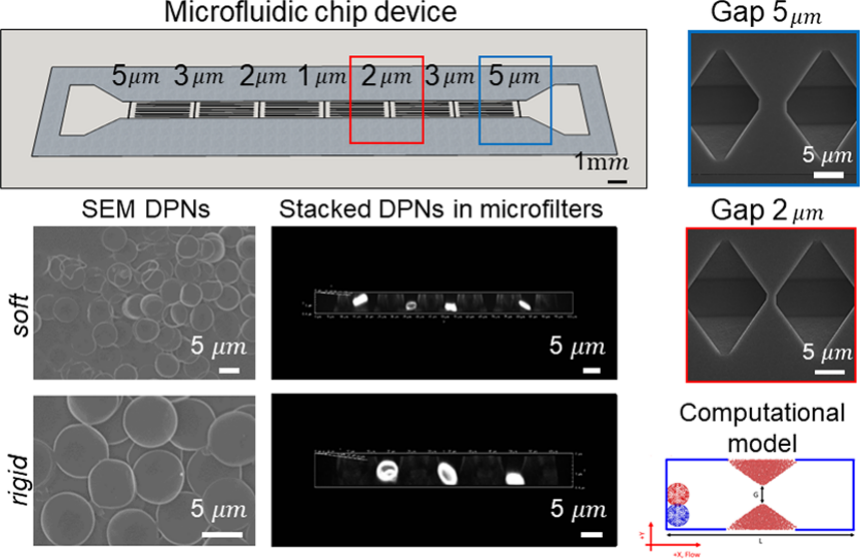

Assessing the mechanical behavior of nano- and micron-scale particles with complex shapes is fundamental in drug delivery. Although different techniques are available to quantify the bulk stiffness in static conditions, there is still uncertainty in assessing particle deformability in dynamic conditions. Here, a microfluidic chip is designed, engineered, and validated as a platform to assess the mechanical behavior of fluid-borne particles. Specifically, potassium hydroxide (KOH) wet etching was used to realize a channel incorporating a series of micropillars (filtering modules) with different geometries and openings, acting as microfilters in the direction of the flow. These filtering modules were designed with progressively decreasing openings, ranging in size from about 5 down to 1 μm. Discoidal polymeric nanoconstructs (DPNs), with a diameter of 5.5 μm and a height of 400 nm, were realized with different poly(lactic-*co*-glycolic acid) (PLGA) and poly(ethylene glycol) (PEG) ratios (PLGA/PEG), namely, 5:1 and 1:0, resulting in soft and rigid particles, respectively. Given the peculiar geometry of DPNs, the channel height was kept to 5 μm to limit particle tumbling or flipping along the flow. After thorough physicochemical and morphological characterization, DPNs were tested within the microfluidic chip to investigate their behavior under flow. As expected, most rigid DPNs were trapped in the first series of pillars, whereas soft DPNs were observed to cross multiple filtering modules and reach the micropillars with the smallest opening (1 μm). This experimental evidence was also supported by computational tools, where DPNs were modeled as a network of springs and beads immersed in a Newtonian fluid using the smoothed particle hydrodynamics (SPH) method. This preliminary study presents a combined experimental–computational framework to quantify, compare, and analyze the characteristics of particles having complex geometrical and mechanical attributes under flow conditions.

## Introduction

In the field of drug delivery, various strategies have been employed to enhance the transport of nano/micron particles at biological target sites. Several independent parameters have been identified that would affect the transport efficiency, including chemical attributes (e.g., composition, surface charge, and targeting moieties);^1,2^ particle physical attributes (e.g., size, shape, and mechanical properties);^3−5^ and biological attributes at the target sites (e.g., expression of specific recognizing receptors, vascular and tissue architectures, cell phenotype, and genetic traits).^6−8^ From an engineering point of view, geometry and mechanical properties are key parameters as they can be modulated during the particle fabrication process. Moreover, while the geometry has been documented to directly impact the particle circulation half-life and tumor accumulation,^9−11^ the role of particle deformability is still under intense scrutiny. Furthermore, a variety of microscopy-based tools and flow-based techniques are currently available to accurately and rapidly characterize particle geometry, while no standardized test has been proposed to assess the mechanical properties of a micro/nanoparticle under flow. Force spectroscopy techniques, such as atomic force microscopy,^12^ optical and magnetic tweezers,^13^ are far from being considered standards as they often provide operator- and machine-dependent results and, importantly, cannot provide quantitative data under flow.

Recently, microfluidics has been explored as a novel approach to address diverse issues ranging from performing on-chip biophysical experiments (lab-on-a-chip)^14^ to modeling complex biological processes,^15^ as well as a strategy to qualitatively examine the mechanical properties of nano- and micron objects.^16^ Indeed, microfluidics allows researchers to modulate, independently, a multitude of hydrodynamic parameters defining unique force, stress, and strain distribution regimens. For instance, the group of Di Carlo studied the role of the geometrical and mechanical properties using inertial microfluidics.^17^ Under relatively low Reynolds numbers, with Re ranging between 1 and 10, this technique defines the spatial position of particles as a function of their size and stiffness. In the same context, another approach was developed by Charrier et al. to determine the deformation mechanisms of healthy and sick red blood cells (RBCs) by forcing them to pass through orifices within a microfluidic channel.^18^ This study laid the basis for the quantification of the rigidity of nonaxial symmetric particles.

Following this line of thought, a microchannel was specifically designed and fabricated via potassium hydroxide (KOH) wet etching to obtain a series of micropillars (filtering modules), aligned orthogonally to the flow, with different opening sizes ranging from about 5–1 μm. Two different discoidal polymeric nanoconstruct (DPN) configurations, having the same geometry but different deformabilities obtained by variating the mixture composition, were considered as model particles.^4^ The passage of DPNs across the series of micropillars was analyzed experimentally by observing their real-time dynamics within the microchannel as well as computationally using the smoothed particle hydrodynamic model to confirm and reinforce the problem at hand.^19−23^

## Materials and Methods

### Fabrication of the Microfluidic Filtering Device

A microfluidic device was designed with a computer-aided design software (Layout-Editor) and realized using a soft lithographic approach. Silicon wafers with surface crystalline plane orientation <110> coated with a 500 nm layer of Ni_3_S_4_ (Si-Mat) were used. Figure S1a shows the orientation of the crystalline planes and the overall microfluidic chip dimensionalities. Figure S1b provides the steps required to realize the microfluidic filtering device during the fabrication process. Before lithography, a uniform layer of AZ 5214 positive resist (Microchem) was deposited over the silicon wafer via spin-coating at 4000 rpm for 1 min. The photoresist was cured on a hot plate at 110 °C for 1 min. The designed microfluidic pattern was transferred on the AZ 5214 positive resist using a Direct Laser Writer system (DLW6000, Heilderberg). Next, to develop the designed pattern, the silicon wafer was immersed in an AZ 726 MF developer (Microchem) for 1 min and then rinsed with water (step 1 of Figure S1b). Before transferring the pattern through the Ni_3_S_4_ layer, an oxygen plasma (O_2_) treatment (Plasma System Tucano, Gambetti) was performed for 120 s at 100 W and 0.5 mBar to clean the surface. The wafer was placed in an inductively coupled plasma-reactive ion etching (ICP-RIE) (SI 500, SENTECH Instruments GmbH, cleanroom facility) to remove the 500 nm layer of Ni_3_S_4_ (step 2 of Figure S1b). Then, a rinse of acetone and isopropyl alcohol (IPA) was performed to remove the AZ 5214 resist, followed by water cleaning and an oxygen plasma treatment, as before (step 3 of Figure S1b). The wet-etching solution was prepared by mixing potassium hydroxide (KOH) (Merck) 30% (w:w), IPA 20% (w:w), and water 50% (w:w). The solution was then placed on a hot plate at 80 °C. The wafer was immersed in the solution for 12.5 min to obtain the final configuration and then rinsed with water (step 4 of Figure S1b). Note that the wet-etching step took place once the solution achieved the set temperature. Then, to remove the Ni_3_S_4_ layer, a second wet-etching step was performed in Ceramic etchant A (Sigma-Aldrich) at 160 °C (Step 5 of Figure S1b). Finally, the surface of the silicon wafer was treated with perfluorotrichlorosilane (ThermoFisher) in a vacuum chamber for 1 h to facilitate the peeling off of the PDMS replica template.

The actual PDMS microfluidic filtering device was realized via soft lithography. First, PDMS (Sylgard 184, Dow Corning) was mixed with the curing agent at a ratio of 4:1 (w:w). Then, it was degassed in a vacuum chamber to remove undesired bubbles. PDMS was poured onto the silicon wafer and cured overnight at 65 °C. After peeling off, the inlet and outlet ports were created with a hole punch. Finally, the microfluidic device was firmly bounded to a glass coverslip (25 mm × 60 mm) for microscopy via a O_2_ plasma treatment (20 s, 20 W, 0.5 mBar).

### Optical Microscopy, Image Acquisition, and Postprocessing

Experiments were performed using an inverted fluorescent microscope (Nikon Eclipse Ti-E) equipped with 63× NA 1.2 and 100× NA 1.2 oil immersion objectives. The microfluidic filtering device was connected to a syringe pump (Harvard Apparatus) with a silanic tube and assembled over the stage of the microscope. An aqueous solution of DPNs (110,000 DPNs/mL) was infused through the microfluidic filter via the syringe pump. The dynamics of DPNs was monitored in real time within the microfluidic filter at four different flow rates (6, 1.5, 0.1, and 0.05 μL/min) for 10 min. After the experiment, the number of adhered particles was counted by acquiring a 3D z-stack (z-gap of 300 nm) for all filter series with 3D confocal microscopy (i.e., Andor camera).

The actual geometry of the microfluidic filter was assessed by flowing a water-based solution carrying the green fluorescent macromolecule 250 kDa Dextran into the PDMS channel. A 3D confocal microscopy reconstruction of the system was performed using an Andor camera. The resulting 3D image was obtained by piecing together 6 × 4 single 3D images corresponding to an overall area of 1.5 × 1 mm^2^.

Videos were acquired using a fluorescent microscope outfitted with a high-speed CMOS camera (Zeyla) using a 63× oil objective. Importantly, reducing the region-of-interest (ROI) by ∼3 times (from 0.25 to 0.1 mm^2^) enhanced the acquisition rate up to 283 frames-per-second (FPS), ∼3 times faster than the nominal frame rate of 101 FPS. The nominal pixel size of the camera was 110 nm.

### Imaging Analysis and Movie Postprocessing

After acquiring multiple images of red fluorescent DPNs under flow using a fast-speed camera, movies were analyzed and postprocessed using MATLAB R2020b. Specifically, the image-processing toolbox and automatic recognition functions (see details below) allowed the detection and geometrical quantification of objects. For the two types of mechanisms, after improving the movie quality (by varying brightness, contrast, saturation), each frame was recursively analyzed and binarized using the built-in MATLAB functions. Further processing employed the use of masks on the binary frames to remove noise and sharply polish objects based on their real morphology. The “regionprops” function then calculates spatial and geometrical features of each recognized object, e.g., “*x*–*y* center coordinates”, “area”, “minor axis”, “major axis”, “perimeter” (see MATLAB code for details). This leads to the investigation of other features such as the DPN dynamics during passage through the filter. The different geometrical characterization performed for sDPNs (axial deformation) and rDPNs (rigid rotation) shared the same image enhancement and noise reduction as described above. For sDPNs, the aim was to quantify the evolution of the major/minor axis ratio over time in order to calculate particle deformation using the “regionprops” function (as discussed above). However, for the rDPNs, quantifying the evolution of the rigid DPN rotation over time was based on the particle fluorescence signal and quantified using the “imgradientxy” function.

### Modeling Particle Transport

DPNs were modeled as an elastic network of beads and springs, whose elastic energy defined as
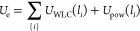
1is implemented through each connected spring in the form
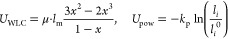
2Here, *U*_WLC_ is a strain-hardening nonlinear restoring energy with *l*_*i*_ being the spring length, and *x* = *l*_*i*_/*l*_*i*_^0^, *l*_m_ = 2.2*l*_*i*_^0^, and *l*_*i*_^0^ is the equilibrium spring length, which is set to be the initial length for each spring. *U*_pow_ provides a repulsive force as the spring is compressed. The *k*_p_ is a dependent variable such that each spring has an energy minimum at *l*_*i*_ = *l*_*i*_^0^. Therefore, only one independent parameter is needed for tailoring the rigidity of the particle—the elastic modulus, μ. Simulated DPNs were immersed in a Newtonian fluid, where the smoothed particle hydrodynamics (SPH) method was adopted to simulate the flow field. The two-way coupling between the SPH-modeled fluid and DPNs was fulfilled including a drag force in the form:
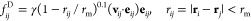
3where γ is the coupling strength and *r*_m_ is the cutoff length. This was set to 0.5 μm, which is about the average spring length of DPNs and is sufficiently long to include multiple surrounding solvent particles. This two-way coupling approximates a no-slip condition at the fluid–DPN interface. For more details regarding the SPH methods, appropriate magnitude of γ, solid boundary conditions near complex surfaces, and the fluid-structure coupling, readers can refer to the literature.^20,24,25^

The computational domain reproduced an area of 50 μm comprising two adjacent micropillars. The pillar and opening geometries were carefully replicated starting from the electron microscopy images of the microchannel, returning a channel height of *H* = 5 μm sandwiched between upper and bottom walls and different gap sizes named as *G* 1.1, 2, 3, and 4.9 μm (corresponding with the actual microfluidic chip). Periodic boundary conditions were applied to the boundaries along the *x* and *y* directions. The upper and lower walls in the computational domain were defined by introducing fixed SPH particles.

In the simulations, the flow was driven by a constant force applied on each solvent particle along the longitudinal *x* direction, mimicking the applied pressure gradients along the channel. Note that each simulation was performed after reaching quasi-steady state flow conditions. As an initial configuration, the center of mass of the DPNs was fixed while shape and velocity were updated at each iteration by the local flow field. At these initial conditions, only negligible shape and orientation changes were observed, simulating the DPN behavior while traveling in the channel under steady conditions.

### Statistical Analysis

ANOVA tests were used to perform the statistical analysis of the data and compare sDPNs with rDPN results passing through various filter modules. The *p*-values of 0.05, 0.01, and 0.001 were identified as *, **, and ***, respectively. Data were presented as the mean ± SD. The *t*-test was performed to compare rDPN and sDPN diameters.

## Results and Discussion

### Geometry of the Microfluidic Filtering Device

**Figure 1:**
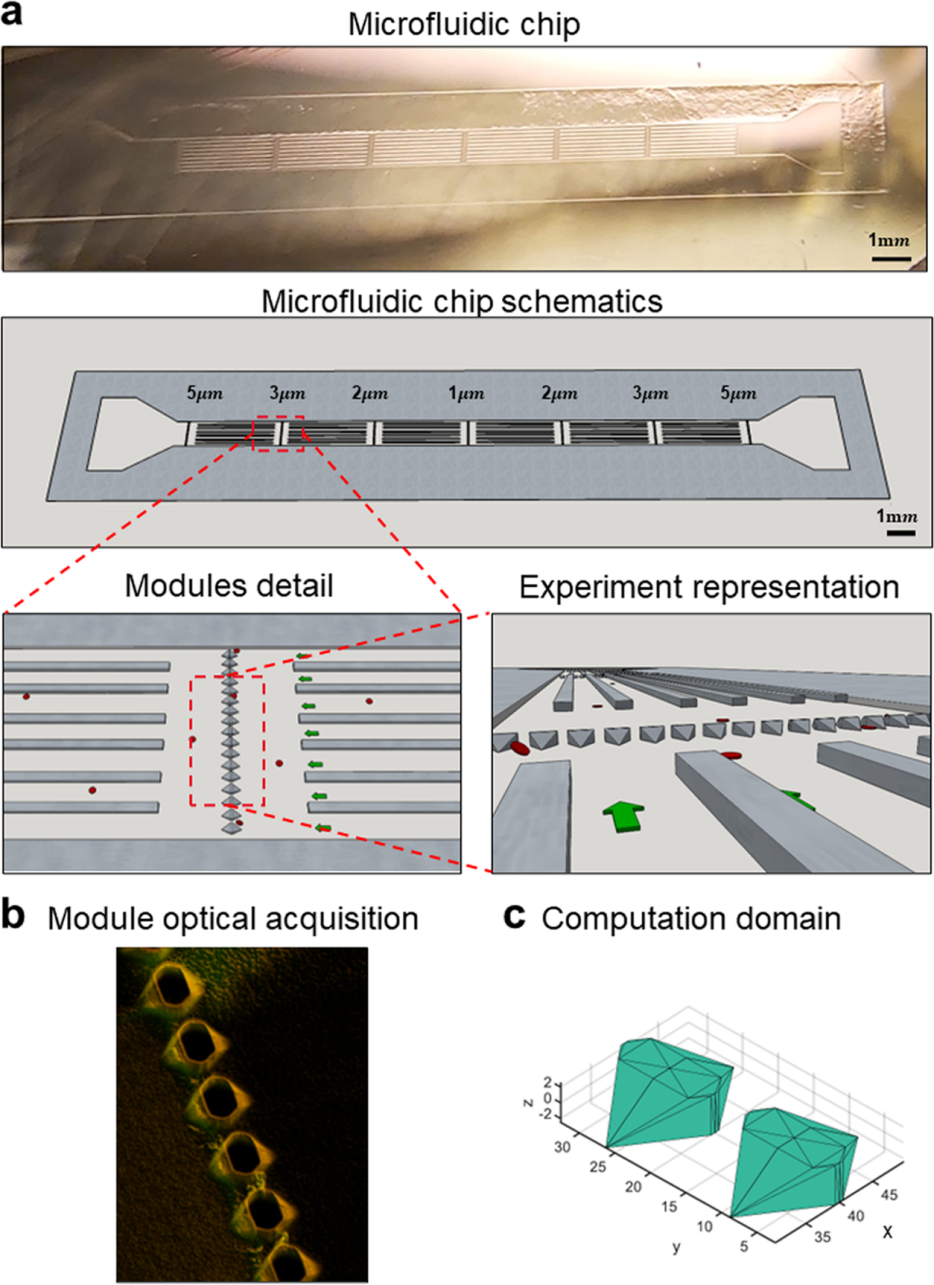
Microfluidic filtering device. a. Image (top) and 3D schematic reconstruction (bottom) of the microfluidic filtering device for assessing particle deformability under flow. The additional two representations at the bottom provide details on the filtering modules and showing the series of micropillars, spanning over the lateral width of the device, the supporting walls, and microparticles (red) transported through the filter openings by the flow. Green arrows indicate the flow *x*-direction. (b) Representative 3D confocal microscopy image depicting a series of micropillars with openings of 5 μm in the microfluidic filtering device. (c) CAD drawing of individual 5 μm opening, documenting the complex design obtained by the KOH wet-etching process in a silicon <110> wafer. This drawing defines the computational domain used for modeling.

The microfluidic device, presented in Figure 1, comprises a single ∼2.0 cm long, 870 μm wide, and 5 μm high channel, with one inlet and one outlet, and 4 series of transversal micropillars constituting the filtering modules with openings of 5, 3, 2, and 1 μm. The microfluidic chip is symmetrically designed, as clearly shown in the image and 3D reconstruction of Figure 1a, so that the inlet and outlet can be readily reversed depending on the needs. The filtering modules are separated from each other in the *x*-direction by 2 mm to prevent any flow disturbance or fluctuation. Moreover, given the high aspect ratio of the channel, having a width/height (870:5) ratio of 160, supporting walls were interposed between sequential filtering modules to ensure structural stability of the soft PDMS replica of the microfluidic chip. Interposing these walls reduces the actual width/height ratio to 15. Importantly, as described in the Materials and Methods section, the lower base/curing agent ratio (4:1) with respect to the standard 10:1 strongly enhances the stiffness of the cured PDMS, thus leading to an enhanced structural stability.^26^ Given the imposed channel height of 5 μm (to prevent any tumbling motion of DPNs), the channel width was selected to be 870 μm to ensure a laminar flow and an effective movie acquisition.

Conventional 2D lithography allows one to realize accurate geometrical patterns at the micron scale but it becomes quite ineffective in the near 1 μm and submicron regime. The characteristic dimensions of the filtering modules and micropillars fall within this regime and, therefore, the anisotropic KOH wet etching of silicon was exploited to realize the device. Specifically, given the different etching rates along the crystalline planes, the KOH wet etching results in a typical 35.26° angle following the <111> (see Figure S1) crystalline planes for the *x*–*y* plane (flow plane). As to the *z* direction, there are two KOH wet etching processes. The first etching process shows an inclination of 35.26° parallel to the <110> crystalline planes, whereas the second etching process progresses perpendicularly to the *x*–*y* silicon plane and to the <110> crystalline planes, as seen in the fluorescence image of Figure 1b, technical drawings of Figures 1c and S2. Thus, the geometry of the microfluidic channel, the openings in the filtering modules, and the supporting walls can be accurately shaped at the micron and submicron scales, knowing the different etching rates between the crystalline planes. Clearly, the morphological variation showed after the KOH wet-etching process (step 4 of Figure S1) needs to be predicted during the microfluidic drawing, as presented in steps 1–3 of Figure S1.

**Figure 2:**
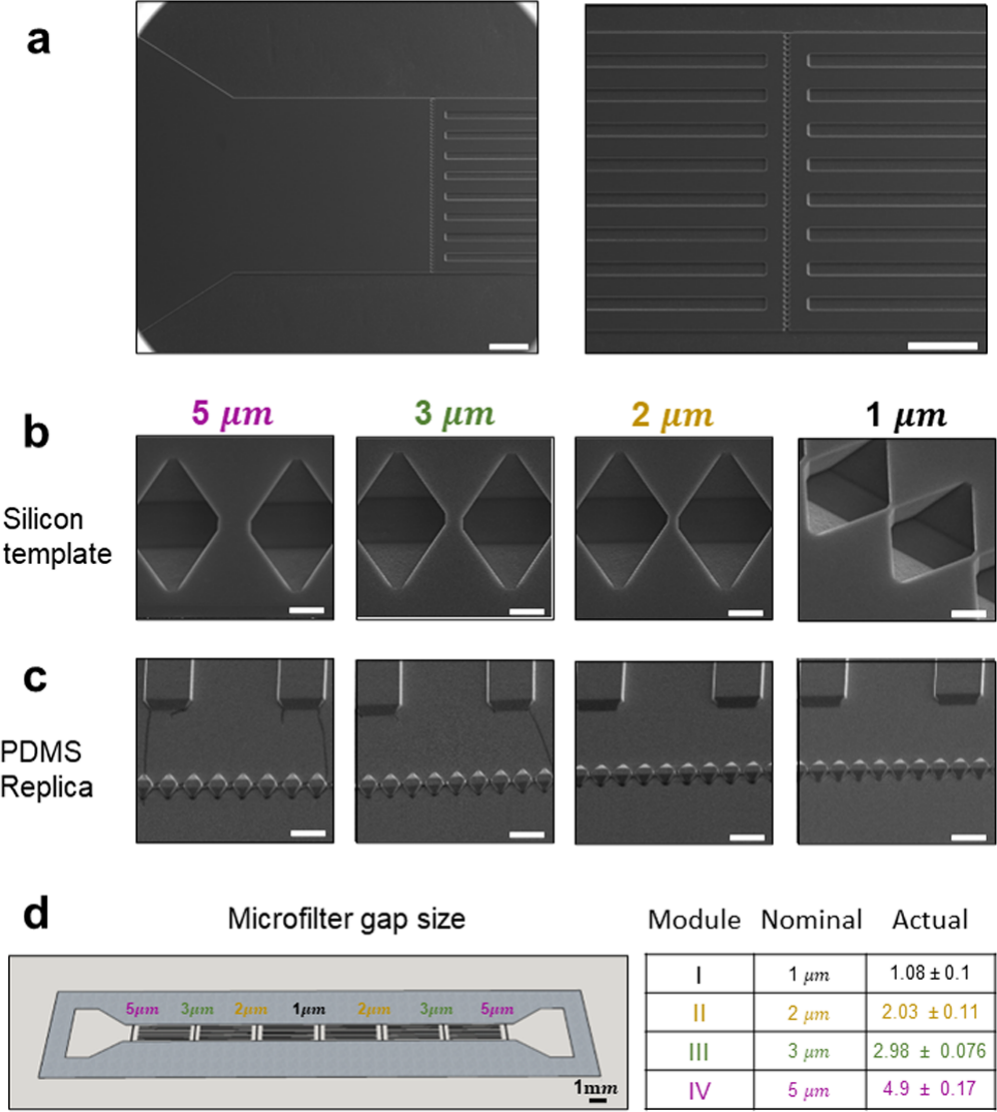
Geometrical characterization of the microfluidic filtering device. (a) Scanning electron microscopy (SEM) images of the silicon template, showing the inlet section (left) and magnified view of a series of microwells (right). Note that upstream and downstream of the microwells, a series of longitudinal microscopic slits equally spaced by 75 μm along the *y*-direction, are interposed (to avoid any structural collapse of the microfluidic channel). The silicon template is replicated into the actual microfluidic filtering device so that the microwells become micropillars and the longitudinal microscopic slits become the supporting walls. Scale bar: 200 μm. (b) SEM images of the microwells in the silicon template for the modules 5, 3, 2, and 1 μm. Scale bar: 5 μm. (c) Corresponding micropillars in the PDMS replica for all four filtering modules 5, 3, 2, and 1 μm. Scale bar: 25 μm. (d) Nominal and actual size of the openings in the four sequential filtering modules.

From the inlet (Figure 2a, left), inflowing particles encounter first the largest filtering module with an opening size of 5 μm, and then the second, third, and fourth filtering modules presenting openings of 3, 2, and 1 μm, respectively. In the silicon template, the micropillars appear as microwells and the supporting walls as longitudinally aligned microscopic slits (Figure 2a, right). Figure 2b shows scanning electron microscopy images of the arrays of microwells in the silicon template, and Figure 2c shows micropillars made of the PDMS replica. The opening size for the 4 sequential filtering modules is detailed in Figure 2c, reporting both the nominal and actual size as derived from multiple measurements (*n* = 17) conducted on the electron microscopy images. Specifically, a nominal opening of 5 μm corresponds to 4.9 ± 0.17 μm; 3 μm corresponds to 2.98 ± 0.076 μm; 2 μm corresponds to 2.03 ± 0.11 μm; and 1 μm corresponds to1.08 ± 0.1 μm.

### Particle Transport along the Microfluidic Filtering Device

To test the filtering performance of the proposed device, soft and rigid discoidal polymeric nanoconstructs (sDPNs and rDPNs) with a diameter of 5.5 μm and a height of 400 nm were realized via a top-down fabrication approach, as described in “Synthesis and Characterization of Discoidal Polymeric Nanoconstructs” (in Supporting Methods). The DPN diameters have been calculated using SEM images and confirmed using a Coulter counter (Figure S3). Importantly, as emphasized in the Coulter counter graph, the size distribution exhibits two distinct peaks at ∼1.4 to ∼2.9 μm. These results are in agreement with our previous works,^8−10^ and the bimodal distribution is due to the nonaxial symmetry of DPNs, as well as the high aspect ratio between the diameter and the height (∼14). Interestingly, the different profiles between soft and rigid DPNs are indicative of the different characteristics regarding deformation under flow conditions (as expected during the Coulter counter measurements) and resulting in a wider distribution for soft DPNs with respect to rDPNs.

**Figure 3:**
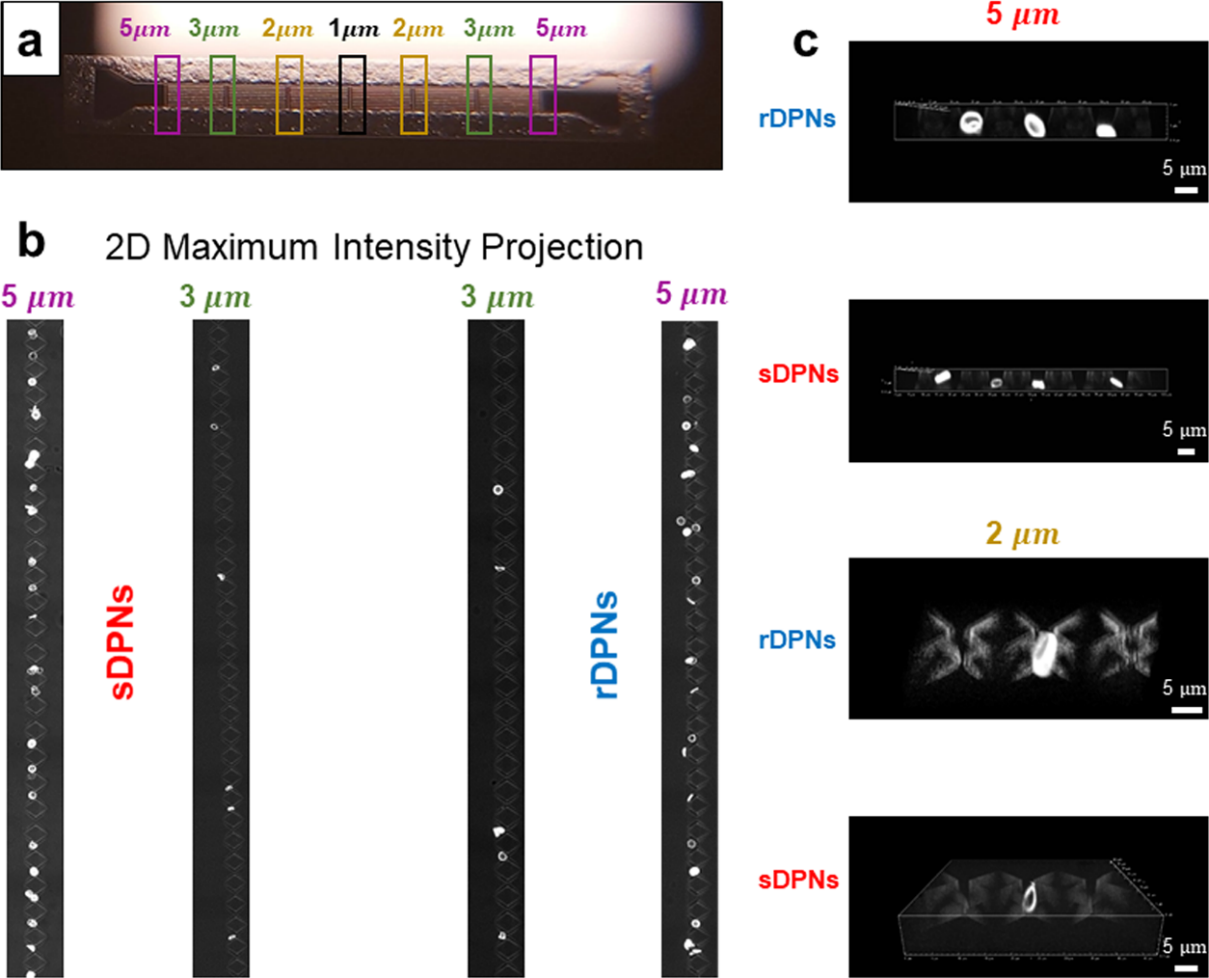
Entrapment of DPNs along the microfluidic filtering device. (a) Confocal microscopy imaging is used to quantify the number of DPNs entrapped at different filter modules along the microfluidic device. (b) Example of 2D maximum intensity projection of confocal microscopy images, showing the accumulation of sDPN (left) and rDPN (right) into 5 and 3 μm filterìng modules for the conditions: particle concentration: 110,000 DPNs/mL; flow rate *Q* = 0.05 μL/min; observation time: 10 min. (c) 3D confocal microscopy images confirming the entrapment of rDPNs and sDPNs in filtering modules of different characteristic size.

These particles were infused in the microfluidic device under controlled conditions, and their ability to deform under flow was assessed indirectly by quantifying the number of DPNs being entrapped in the four different filtering modules. Indeed, only the most deformable particles would be able to squeeze and cross all of the filtering modules from the largest (5 μm) to the smallest (1 μm). An aqueous solution containing a fixed concentration of particles (110,000 DPNs/mL) was prepared and infused for 10 minutes through the inlet port into the microfluidic device (Figure 3a) at four different flow rates, namely, 6.0, 1.5, 0.1, and 0.05 μL/min. Note that the DPN concentrations were carefully calibrated not only to rapidly clog the filtering modules but also to provide enough events (entrapped particles) during the observation period (10 minutes) to draw statistically relevant conclusions. At the end of the experiments, 3D images were acquired at the filtering modules. Figure 3b shows the 2D maximum intensity projection of those 3D images, allowing the quantification of the entrapped DPNs. Figure 3b provides a representative image, where sDPNs and rDPNs (white spots) appear entrapped between pillars (light gray spots) in 5 and 3 μm filtering modules. Examples of entrapped DPNs are presented in Figure 3c. The pictures show different DPN configurations after being entrapped in the microfilters, demonstrating a more pronounced tilting for smaller opening gaps. This is in accordance with the principle of minimum energy in which the DPNs find specific configurations to overcome the obstacles, minimizing the dissipation of energy. Indeed, the DPN configuration evolution in the microfilter consists of a fine balance between orientational changing and deformation. Figure 3 unveils these mechanisms and, qualitatively, allows one to differentiate between the two DPNs (soft and rigid).

**Figure 4:**
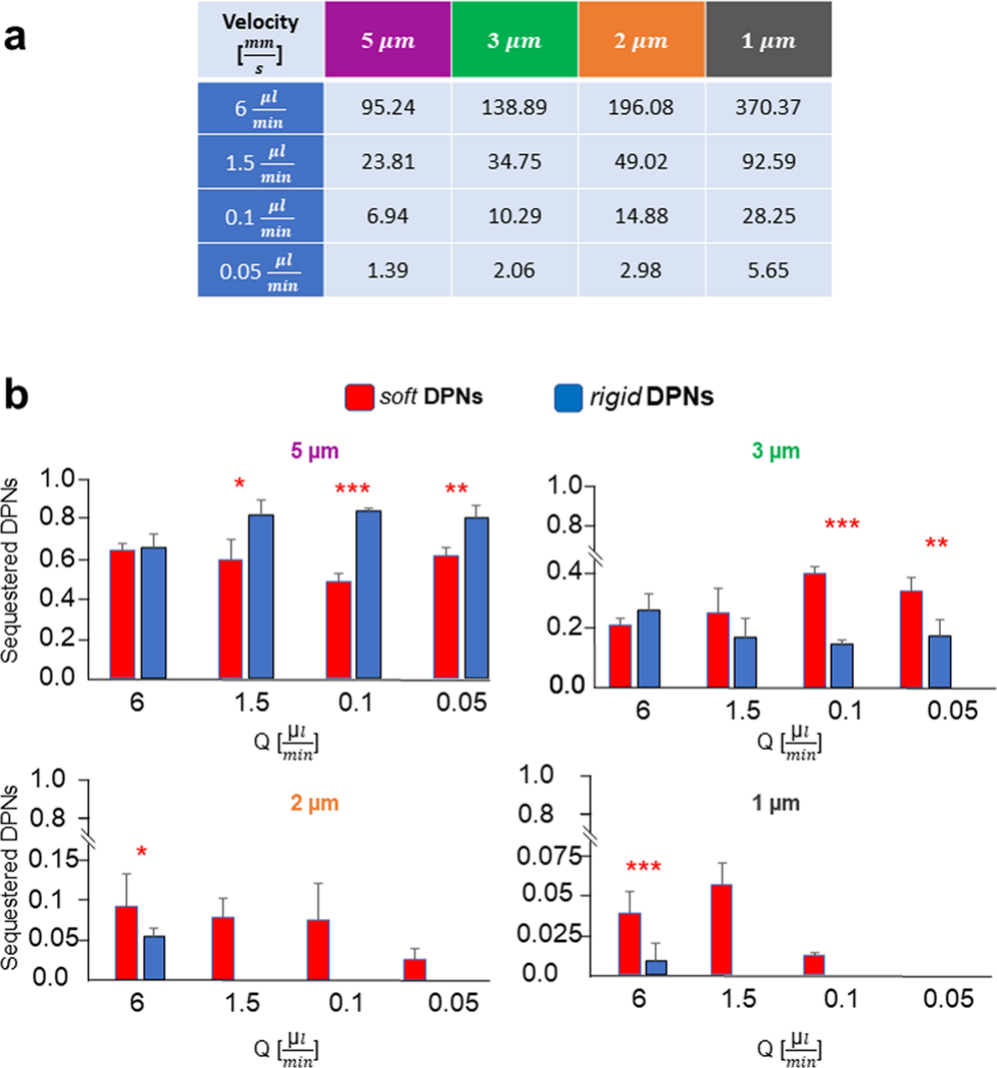
Quantification of DPN entrapment in different filtering modules. (a) Average fluid velocity at different filtering modules and flow rates *Q*. (b) Number of DPNs entrapped at the different filtering modules normalized by the total number of entrapped DPNs along the microfluidic device as a function of the flow rate. Note that the vertical scale of the bar charts changes with the filtering module.

For each of the given flow rates, the average fluid velocity in the opening sections increases moving from the larger to smaller filtering modules, as listed in the table of Figure 4a. Specifically, the average fluid velocity increases from 1.39 mm/s for the lowest flow rate and largest opening (0.05 μL/min and 5 μm) to 370.37 mm/s for the highest flow rate and smallest opening (6 μL/min and 1 μm). Note that these flow velocity values are comparable with the average blood velocity in arterioles, venules, and large vessels. The local flow conditions are expected to affect the particle deformation and its ability to stretch and pass through the opening. From confocal microscopy images, the number of DPNs accumulating within each filtering module can be readily calculated and normalized by the total number of DPNs being trapped in the filter. These data are conveniently presented in Figure 4b for the four different filtering modules (5, 3, 2, and 1 μm), four different flow rates (6.0, 1.5, 0.1, and 0.05 μL/min), and two DPN configurations (rDPNs—blue bars; sDPNs—red bars). As expected, the charts immediately project the information that sDPNs can reach the last two filtering modules (2 and 1 μm) more abundantly than rDPNs. Also, except for the highest flow rate (6 μL/min), only a few rDPNs were trapped in the last two filtering modules, whereas no rDPNs were able to reach these modules for lower flow rates. Similarly, in the largest filtering module (5 μm), at the lowest velocities, rDPNs were entrapped more than sDPNs. Furthermore, for all DPNs, a progressive decrease in the number of entrapped particles per filtering module was documented moving from the largest to the smallest openings (i.e., in the direction of flow). These results unequivocally demonstrate the role of particle deformation and local flow conditions in favoring or opposing the crossing of filtering modules: rDPNs were mostly (>70%) entrapped in the first filtering module while only a modest 1% could reach the smallest openings at the highest flow rate; in contrast, over 50% of sDPNs was able to cross the first filtering modules at moderate and low flow rates.

### Modeling Particle Transport along the Microfluidic Filtering Device

**Figure 5:**
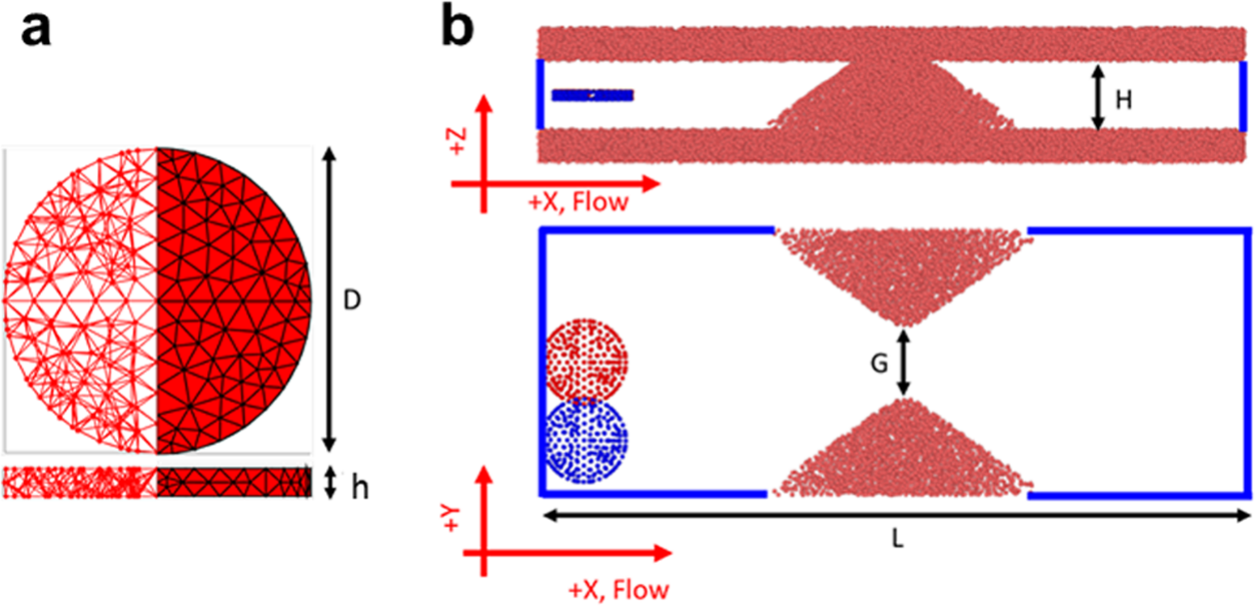
Modeling the dynamics of discoidal polymeric nanoconstructs (DPNs) across a filtering module. (a) Elastic network comprising beads and strings reproducing the geometrical and mechanical properties of DPNs. At rest, DPNs have a diameter *D* = 5.5 μm and thickness *h* = 0.4 μm. The network of elastic strings constituting the structure of DPN is shown in the 3D transparent view (left) and surface view (right). (b) Computational domain reproducing an area within two adjacent micropillars, with a total length in the *x*-direction (flow direction) of *L* = 50 μm. Periodic boundary conditions are applied along *x* and *y* (blue boundary lines). Initial DPN configuration centered (in red) and off-centered (in blue) with respect to the center of the filtering module.

**Figure 6:**
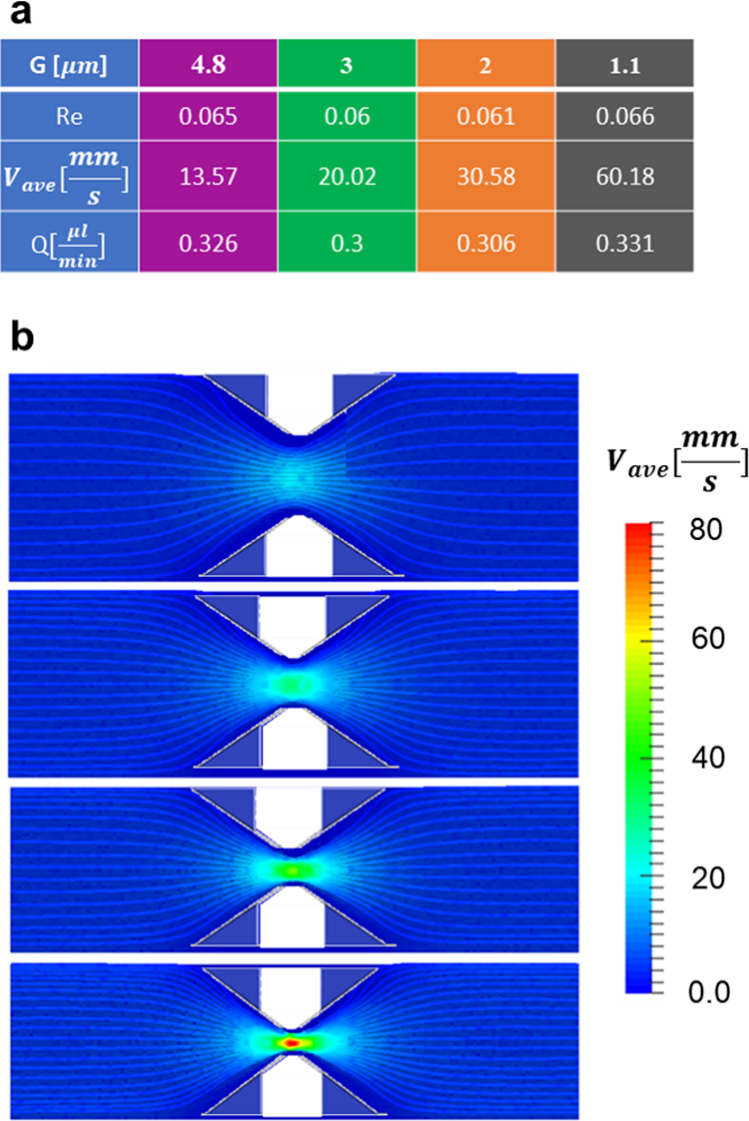
Flow conditions at the filtering modules for *Q* = 0.3 μL/min. (a) Reynolds number (Re≡ρGv_ave_/η), average flow velocity (*v*_ave_), and flow rates (*Q*) for the four different filtering modules are listed in the table. Fluid density (ρ = 1000 kg/m^3^) and dynamic viscosity (η = 1 mPa/s^–1^) were considered for the simulations. (b) Flow field and streamlines on the *x*–*y* flow plane at steady state, in the absence of DPNs, for the four different filtering modules.

A smooth particle hydrodynamic (SPH) model was used to predict the behavior of DPNs passing through the filtering modules. DPNs were modeled as a network of springs and beads; the independent parameter μ characterizes spring stiffness and thus affects the overall particle deformability (Figure 5a). The computational domain was reproduced by referring to the electron microscopy images of the microwell (Figure S3). Also, for the initial conditions, two configurations for DPNs were considered; the first with the particles centered within the channel (red) and the second with the particles off-center (blue) with respect to the opening axis (Figure 5b). Experimentally, the major differences in rDPN and sDPN entrapment occur at moderate flow rates (Figure 4b). For the computational analysis, a fixed pressure drop was considered mimicking the experiments having a flow rate *Q* ∼ 0.3 μL/min. At this imposed pressure drop, the fluid dynamic parameters (i.e., Reynolds, average velocity, and flow rate) are presented in the table of Figure 6a. In the absence of particles, the steady-state velocity field *v*_*x*_ is presented in Figure 6b for the four openings. The flow field is symmetric, and the streamlines away from the filter modules are aligned to the *x*-axis, indicating that the periodic boundary conditions are properly imposed.

**Figure 7:**
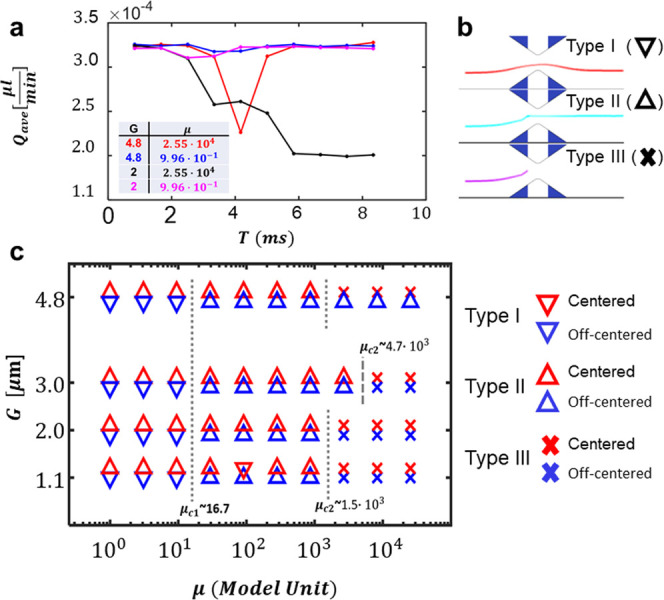
Predicting the DPN behavior across different filtering modules. (a) Flow rate variation across *G* = 5 and 2 μm openings, while soft (μ = 9.66 × 10^–1^) and rigid (μ = 2.56 × 10^4^) DPN are passing throughout. Line colors follow the legend embedded in the figure. (b) Three types of trajectories are identified for DPNs crossing the filtering modules: crossing by following the streamlines with no blockage (type I, ∇) (μ = 10, *G* = 5 μm); crossing by causing a temporary blockage (type II, Δ) (μ = 2.95 × 10^2^, *G* = 5 μm); crossing by causing a permanent blockage (type III, x) (μ = 2.56 × 10^4^, *G* = 5 μm). (c) State diagram for various DPN deformability values (μ = 10–104) and opening sizes (1–5 μm) (blue symbols—initially off-centered DPNs, red symbols—initially centered DPNs).

The presence of DPNs is expected to significantly affect the flow conditions at the openings. Depending on the size ***G*** of the openings as well as the particle deformability **μ** and its orientation, different scenarios could be anticipated: some particles could readily cross the filter (type I) as opposed to other particles that would occlude the filter either transiently (type II) or permanently (type III). These three different behavior types can be immediately linked to the flow rate variation associated with the passage of a single rigid (rDPN – μ = **2.55 × 10**^**4**^) or soft (sDPN – μ = **9.96 × 10**^**–1**^) particle. For soft particles, no significant alterations in flow rate were observed as sDPNs deform and rotate and rapidly cross both the large 5 and small 2 μm openings without inducing any obstructions—type I (Figure 7a, blue and pink lines; Figure 7b, ∇). For rigid particles, a transient blockage is observed when moving across a 5 μm opening, causing a temporary drop in flow rate from **3.25 × 10**^**–4**^ to **2.25 × 10**^**–4**^ μL/min that is fully recovered after particle passing—type II (Figure 7a, red line; Figure 7b, Δ). However, if the same rigid particle approaches a 2 μm opening, as expected, a permanent blockage is induced leading to a definitive drop in flow rate to **2.25 × 10**^**–4**^ μL/min—type III (Figure 7a, black line; Figure 7b, X). This behavior depends not only on the relative particle-to-opening size and particle deformability but could also be affected by the initial particle position within the flow field. As such, a simulation campaign was conducted for multiple ***G*** and **μ** values and initial particle locations returning the map of Figure 7c, where the red (blue) symbols are related to DPNs initially centered (off-centered); downward (upward) pointing triangles are referred to passing (temporally blocked particles), whereas crosses are related to permanently blocked particles (legend of Figure 7c). Sufficiently, soft particles (**μ ≲ 15**) crossed filter modules of any size (left portion of the map in Figure 7c). However, initially, off-centered particles (blue symbols) would cause less flow alterations than initially centered particles (red symbols), as the former would rotate in addition to deform, and thus, a more efficient passing through modules was detected. At the other extreme, rigid particles (**μ** ≳ **2×10**^3^) did not cross openings smaller than about 3 μm. Interestingly, however, rigid particles crossed 5 μm openings only if initially were off-centered, as this would help them orient properly (see upward pointing blue triangles as opposed to red crosses in the top right corner of the map in Figure 7c). Finally, type I and mostly type II behaviors are depicted in the center of the map for intermediate particle deformability values.

### Particle Dynamics across Filtering Modules: Computational Predictions and Experimental Validations

**Figure 8:**
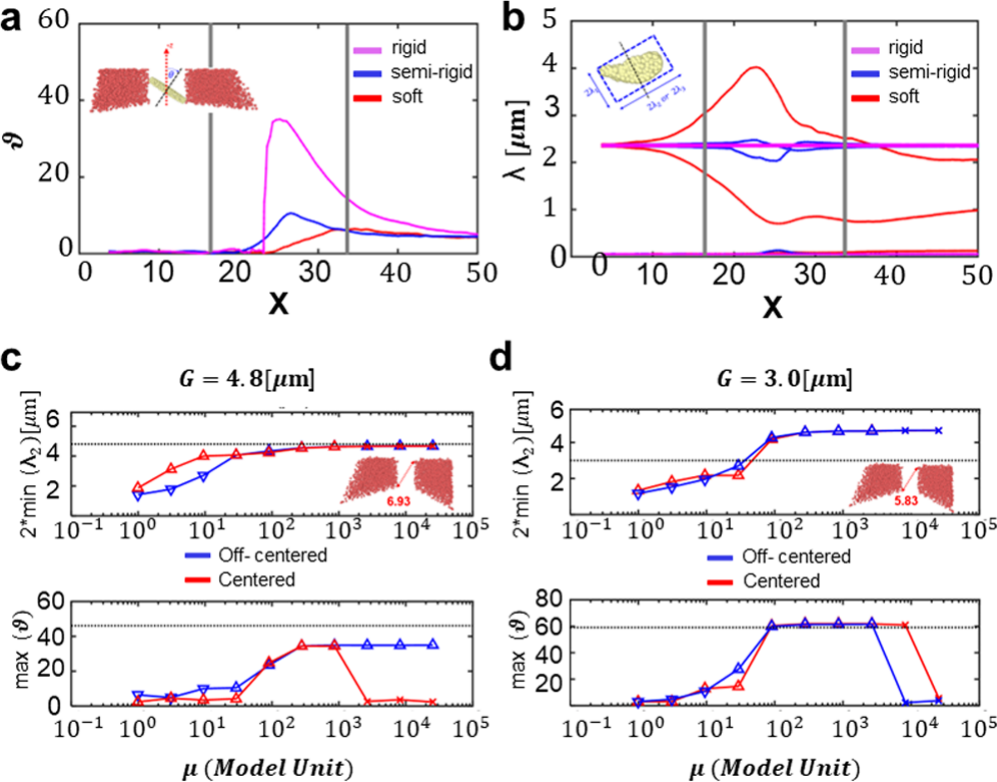
DPN rotation and deformation in crossing the filtering modules. (a) Variation of the orientation θ angle of DPNs while crossing a 5 μm filtering module. (b) Variation of the eigenvalues λ_1_, λ_2_, λ_3_ of the elasticity tensor of DPNs while crossing a 5 μm filtering module (pink line—μ = 2.55 × 10^4^; blue line—μ = 30; red line—μ = 0.9). The gray lines indicate the start and end sections of the filtering module along the flow *x*-direction. (c, d) Minimum width (2 min (λ_2_)) and maximum angle of rotation (max (θ)) of DPNs crossing 5 μm (c) and 3 μm (d) openings. Red (blue) lines are for initially centered (off-centered) DPNs. The symbols ∇, Δ, and *x* represent type I, II, and III crossing behaviors. The black dotted line indicates the opening size G and maximal flip angle θ = atan(G/h), respectively.

To further characterize the DPN dynamics, the particle orientation and deformation were monitored during the crossing of the filtering module. The orientation of the particle was defined through the angle θ between the *z*-direction and the axis of symmetry of the discoidal DPNs (Figure 8a), while the deformation of the particle was assessed considering the values of the three eigenvalues λ_1_ ≤ λ_2_ ≤ λ_3_ of the inertial tensor (Figure 8b). In Figure 8a,b, the variation of the particle orientation θ and deformation parameters λ is plotted along the flow direction *x*, respectively. In both plots, the gray bars identify the start (*x* = 15) and the end (*x* = 35) sections of the filtering module. For a 5 μm opening, rigid DPNs (μ = 2.55 × 10^4^) moving from an off-centered initial position rotated by almost 40 degrees in order to cross the filtering module (pink line in Figure 8a) and, given the rigidity of the particle, no deformation was observed (pink lines in Figure 8b). This is a type II behavior and clearly demonstrates how 5.5 μm rigid particles could cross a 5 μm opening only if properly oriented. Note that a 5 μm opening has a diagonal gap size of almost 7 μm, which significantly exceeds the resting size of a DPN.

However, soft particles (μ = 0.9) did not significantly rotate while approaching the filter (red lines in Figure 8a) but underwent a compression in the *y*-direction (λ_2_) and elongation in the *x*-direction (λ_3_) (red lines in Figure 8b). This is a type I behavior. For particles with an intermediate stiffness modulus (μ_c1_ = 16.7 < μ < μ_c2_ = 2.55 × 10^4^), namely, types I and II, we assist to the coexistence of the two mechanisms; therefore, orientation and deformation cannot be decoupled (μ = 30—blue lines in Figure 8a,b). In Figure 8c,d, DPN rotation and deformation were assessed for different deformability values and filter opening sizes, being *G* = 5 and 3 μm, respectively. These data confirm that rigid particles can only cross the larger opening by rotating (Figure 8c); soft particles preferentially deform rather than rotating (left side of the plots in Figure 8c,d), while off-centered particles are more prone to change their orientation and rotate (blue lines in Figures 8c,d and S4).

Experimentally, capturing the actual deformation and rotation of a DPN crossing a series of micropillars is extremely challenging given the geometry, flow conditions, and time constant of the problem at hand. Two Supporting Movies document the typical dynamics of sDPNs and rDPNs crossing a 5 μm filtering module, whereby the first tends to undergo to a deformation only and the second to orientation only mechanism. In the case of soft particles, Figure S5a details the ratio between the major and minor axes of an sDPN while traversing a 5 μm filtering module. For most of the observation time, the ratio is close to unity given the circular shape of the DPNs. However, for a tiny fraction of time corresponding to ∼0.05 s, the ratio rapidly grows to ∼1.25 and then decreases back again to 1. In the case of rigid particles, Figure S5b documents a rotation of the particle as suggested by the change in fluorescence intensity associated with the tilted as opposed to the horizontally laying particle. Following the variation in fluorescence signal associated with rotating rDPN, a maximum inclination of about 40° was quantified, which is in striking agreement with the computational values of Figure 8a.

Overall, these results confirm that sDPNs as opposed to rDPNs would significantly deform and cross even small openings under different flow conditions.^4,27^

## Conclusions

In this study, the mechanical properties of microparticles with a nonspherical shape were assessed under dynamic conditions using an ad-hoc designed microfluidic chip integrating multiple sequential filtering modules. A microfluidic filtering device presenting openings ranging from 5 to 1 μm was realized by finely controlling the KOH wet etching of silicon wafers, followed by conventional replica-molding techniques. The dynamics and mechanical behavior of 5.5 μm discoidal particles was experimentally determined within the microfluidic filtering devices and computationally modeled via a smooth particle hydrodynamic model. Experiments were conducted considering soft and rigid particles at four different controlled flow rates. sDPNs only were able to reach the smallest filtering modules, whereas the majority of the rDPNs were entrapped in the first module. Modeling allowed the authors to identify different particle behaviors in crossing the filter openings based on their initial location, deformability, and opening size, including the deformation of the particle without obstruction, temporary blocking, and permanent blocking. Interestingly, particles off-center from the filter openings experience hydrodynamic forces that would facilitate their rotation and crossing of the opening, even in the case of rigid particles nominally larger than the orifice. This study offers a computational–experimental framework for testing the mechanical behavior of microparticles under authentic flow conditions.

## Supporting Information Available

Methods of DPN fabrication; DPN characterization; pictures depicting details of microfluidic chip fabrication, microfilter geometry characterization, and design of computational domain; experimental results with MATLAB video postprocessing of DPN deformation and rotation under flow; additional modeling results (PDF)
