# Supplementary material for: Assessing Differential Particle Deformability under Microfluidic Flow Conditions
Source: ACS Biomater Sci Eng. 2023 May 17;9(6):3690–8. doi: 10.1021/acsbiomaterials.3c00120 (PMC10265569; doi:10.1021/acsbiomaterials.3c00120)
Supplement: Supplementary file 1 — ab3c00120_si_001.pdf [file ab3c00120_si_001.pdf]

Supporting Information

ASSESSING DIFFERENTIAL PARTICLE DEFORMABILITY UNDER MICROFLUIDIC FLOW CONDITIONS

Marco E. Miali,[a,Θ] Wei Chien,[a,b] Thomas Lee Moore,[a] Alessia Felici,[a] Michele Oneto,[a] Dmitry Fedosov,[b] Paolo Decuzzi[a]

[a] Laboratory of Nanotechnology for Precision Medicine, Fondazione Istituto Italiano di Tecnologia, Via Morego 30, 16163 Genoa, Italy

[b] Institute of Biological Information Processing, **Forschungszentrum Jülich GmbH**, Wilhelm-Johnen-Straße, 52428 Jülich, Germany

Θ corresponding author: Marco E. Miali Ph.D., marco.miali@weizmann.ac.il

SUPPORTING METHODS

Synthesis and Characterization of Discoidal Polymeric Nanoconstructs. Soft and rigid discoidal polymeric nanoconstructs (sDPNs and rDPNs) with a diameter of 5.5 μm and a height of 400 nm were realized *via* a top-down fabrication approach.[1, 2] The deformability of DPNs was modulated by mixing the polymeric constituents (PEG and PLGA) at different ratios. Briefly, an increase in the PEG content in the polymer mixture softens the DPNs, whereas a higher PLGA content provides rigid DPNs. Importantly, by using a top-down approach, the rigid (rDPNs) and soft (sDPNs) DPNs essentially had the same geometry but different deformability. The morphology of DPNs was characterized by scanning electron microscopy (SEM) (**Figure S2a**) and via a Beckman Mulstisizer 4e Coulter counter (**Figure S2b**). The SEM analyses conducted on 17 samples documented an average diameter of 5,470 ± 140 nm for sDPNs and 5,540 ± 150 nm for rDPN, with no statistically significant difference (p-value = 0.2783). Given the non-spherical DPN shape, the Beckman Mulstisizer 4e Coulter counter returns a size distribution spectrum whose maximum can be generally associated with the characteristic particle size (**Figure 3b**). Notably, while the rDPNs (blue) presented a clear and sharp primary peak around 1,800 nm followed by a secondary peak at around 2850 nm, sDPNs (red) showed a larger peak with a maximum around 1440 nm and a very moderate secondary peak (2770 nm). This very different size distribution spectrum has to be ascribed to the different deformability of the particles, whose size in the counter system is assessed under continuous flow in a 20 μm capillary tube.

SUPPORTING RESULTS AND FIGURES

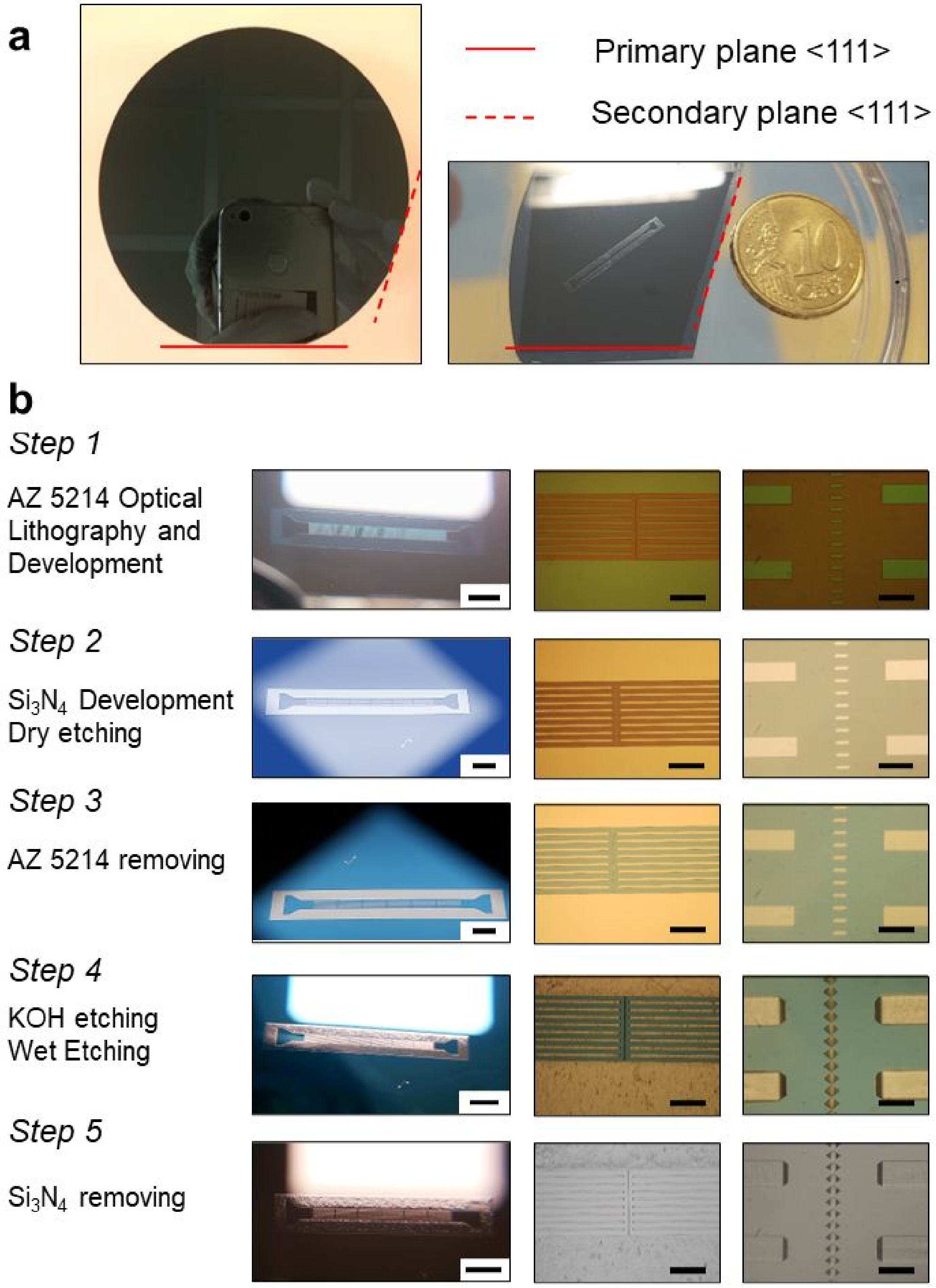

S1. Fabrication process of the microfluidic filtering device. a. Crystalline <110> plane orientation with respect to the silicon wafer (*left*) and microfluidic chip (*right*). **b.** Fabrication steps: *Step 1* – AZ 5214 positive photoresist exposure and development; *Step 2* – ICP-RIE dry etching to remove the Si_3N_4 layer and transfer the pattern on the silicon wafer; *Step 3* – AZ 5214 positive photoresist removal; *Step 4* – KOH wet etching to achieve the final designed configuration; *Step 5* – Ceramic wet etching to fully remove the Si_3N_4 layer. From left to right, the images show the same sample at progressively higher magnifications. The scale bars are 2 mm, 500 µm, 50 µm for the first, second and third column respectively.

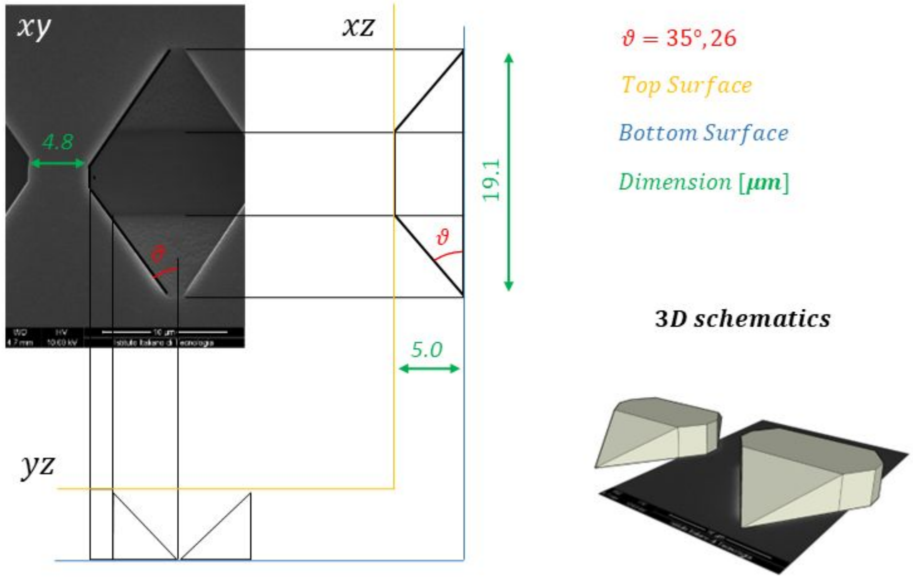

S2. 2D lateral view perspectives and 3D schematic reconstruction obtained from scanning electron micrographs of the silicon template.

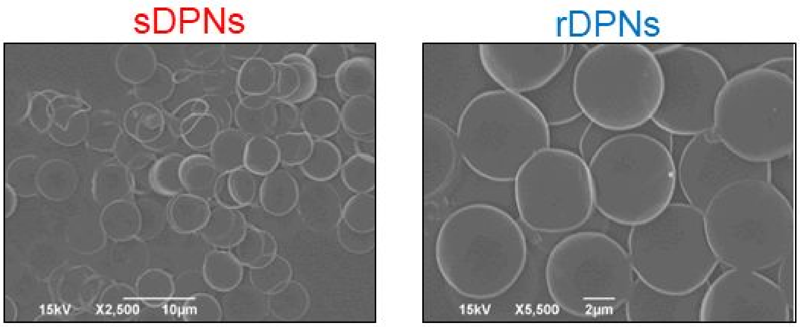

Nanoconstruct	Diameter [μm]
sDPN	5.47±0.14
rDPN	5.54±0.15

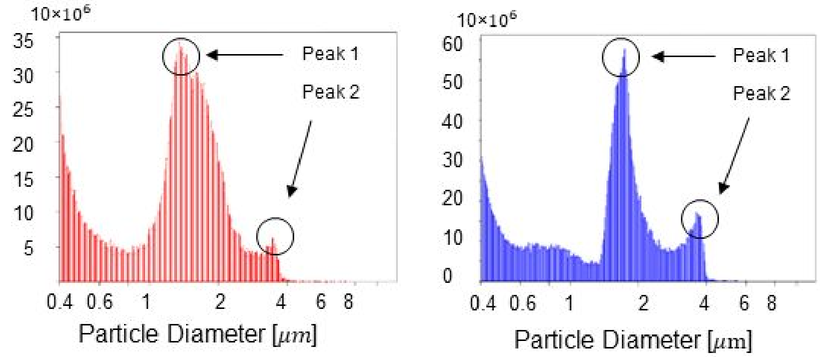

Nanoconstruct	Peak **1**, Peak **2** [μm]
sDPN	**1.44, 2.77**
rDPN	**1.8, 2.85**

S3. Discoidal polymeric nanoconstructs (DPNs) morphological characterization. a. SEM images of soft (sDPN) and rigid (rDPN) particles presenting a diameter of 5.5 µm and height of 0.4 µm. Size distribution (mean ± std) for the two DPN configurations was calculated from multiple SEM images (n = 17). **b.** Size distribution from a Multisizer Coulter counter analysis for sDPN and rDPN. The values of the primary and secondary peaks are listed in the table for the two DPN configurations.

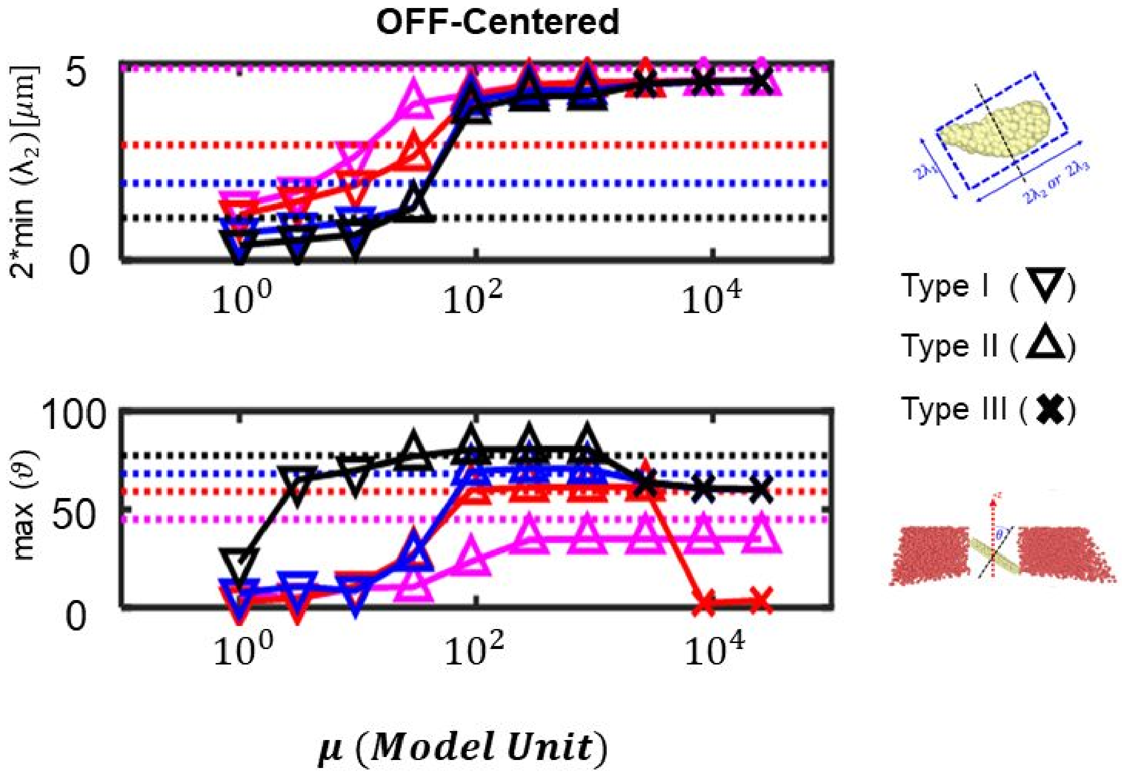

S4. DPNs deformation and rotation for off-centered initial conditions over the elastic modulus μ. The minimum width $(2 \cdot min\,(\lambda_2))$ and maximum angle of rotation $(max\,(\theta))$ of DPNs crossing a 5 μm (pink), 3 μm (red), 2 μm (blue), and 1 μm (black). The symbols ∇, △, and x represent *type I, II* and *III* crossing behaviors. The dotted-lines indicate the opening size G (upper graph) and the maximal flip angle θ = atan(G/h) (bottom graph) respectively. All DPNs start from off-center locations.

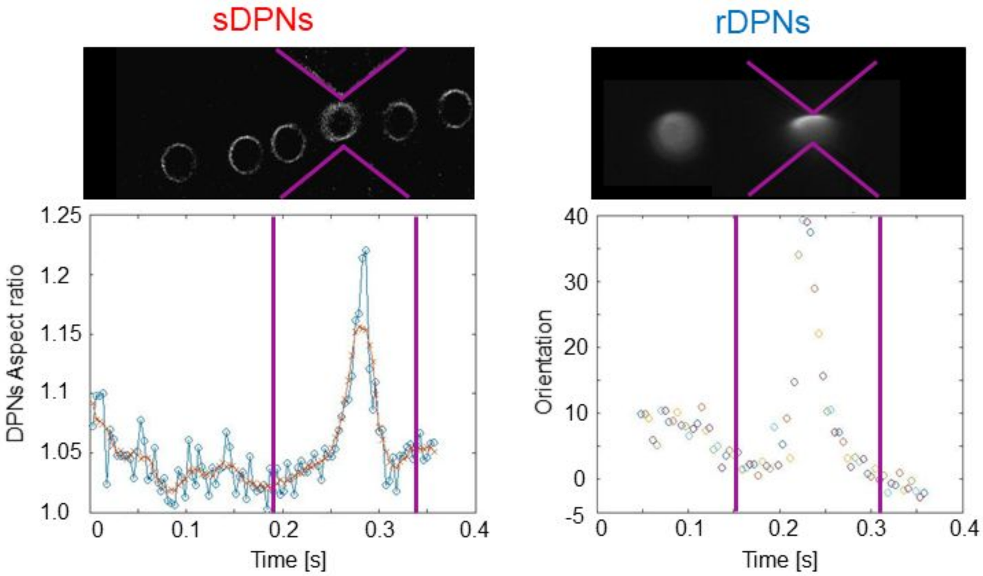

S5. DPN radial deformation and rotation while crossing a 5 µm filtering module. a. Sequential images of a sDPN crossing a 5 µm filtering module. **b.** Major-to-minor DPN axis ratio as crossing the filtering module demonstrating a contraction of sDPN in the lateral direction and an extension along the flow direction (Q = 0.05µl/min). **c**. Images of a rDPN before entering and inside a 5 µm filtering module, showing the change in fluorescent intensity as ascribed to a particle rigid rotation within the field of view. **b.** rDPNs rotation over time as approaching and crossing a 5 µm filtering module.

SUPPORTING MOVIES

Supporting Movie 1. Dynamics of a rDPNs through a 5 µm opening. rDPNs_movie
rDPNs were infused from the inlet port of the microfluidic chip at a flow rate of Q = 0.05 µl/min. In the movie, a rigid DPN is successfully crossing a 5 µm opening. The 280-fps acquisition rate used for generating this movie allowed to precisely capture the trajectory of the rDPN and its inclination, based on the fluorescent intensity distribution over time. As the rDPN approaches the opening in the microfilter, the uniformly distributed fluorescence changes as a small portion of the particle moves out from the focal plane justified by the particle rotation. After crossing the opening, the rDPN rotates back to the original horizontal inclination following the channel streamlines.

Supporting Movie 2. Dynamics of a sDPNs through a 5 µm opening. sDPNs_movies

sDPNs have been flowed from the inlet port of the microfluidic chip at a flow rate of Q=0.05 μl/min (as for the rDPNs). In the movie, the microfilter series is describing a soft particle successfully passing a 5 μm opening. The fps (280) used for the acquisition precisely described the trajectory followed by the sDPNs and, studying the fluorescent intensity, the mechanism of radial deformation is quantified. The sDPNs shows a perfect circularity (i.e. horizontality) whilst travelling in the main channel and leading to a symmetric deformation nearby the microfilter region and demonstrating a *pure* radial deformation mechanism with the absence of any rotational effect.

SUPPORTING REFERENCES

1. Palange, A. L.; Palomba, R.; Rizzuti, I. F.; Ferreira, M.; Decuzzi, P. J. M. T., Deformable discoidal polymeric nanoconstructs for the precise delivery of therapeutic and imaging agents. **2017,** *25* (7), 1514-1521.
2. Key, J.; Palange, A. L.; Gentile, F.; Aryal, S.; Stigliano, C.; Di Mascolo, D.; De Rosa, E.; Cho, M.; Lee, Y.; Singh, J. J. A. n., Soft discoidal polymeric nanoconstructs resist macrophage uptake and enhance vascular targeting in tumors. **2015,** *9* (12), 11628-11641.
